# Synthesis and Characterization of Silibinin-Loaded ZIF-8 Nanocomposites and Their Antibacterial Activity Against *Escherichia coli*: Insights into acrB, recA, and htpX Gene Expression

**DOI:** 10.3390/antibiotics15100951

**Published:** 2026-09-24

**Authors:** Başak Bedir, Mehmet Ersatir, Mehmet Çimentepe, Seval Irem Iyikan, Akın Yiğin, Metin Yildirim

**Affiliations:** 1 Department of Nutrition and Dietetics, Faculty of Health Sciences, Hakkari University, Hakkâri 30000, Türkiye; 2 Department of Chemistry, Faculty of Art and Science, Cukurova University, Adana 01330, Türkiye; 3 Department of Pharmaceutical Microbiology, Faculty of Pharmacy, Harran University, Sanliurfa 63300, Türkiye; 4 Department of Pharmaceutical Chemistry, Faculty of Pharmacy, Cukurova University, Adana 01330, Türkiye; 5 Department of Genetics, Faculty of Veterinary Medicine, Harran University, Sanliurfa 63200, Türkiye; 6 Department of Biochemistry, Faculty of Pharmacy, Cukurova University, Adana 01330, Türkiye

**Keywords:** silibinin, ZIF-8, *Escherichia coli*, nanocomposites, gene expression

## Abstract

**Background/Objectives:** In this study, ZIF-8 and silibinin-loaded ZIF-8 nanostructures containing different concentrations of silibinin were successfully synthesized using a one-pot method and characterized by FT-IR, SEM, and EDX analyses. **Methods:** The antibacterial activities of the synthesized nanostructures were subsequently evaluated against *Escherichia coli*, *Escherichia coli* O157:H7, *Salmonella Typhimurium*, and *Listeria monocytogenes* using the minimum inhibitory concentration (MIC) and minimum bactericidal concentration (MBC) assays. **Results:** Among all tested formulations, S2@ZIF-8 exhibited the strongest antibacterial activity against *E. coli*, with an MIC of 32 μg/mL and an MBC of 64 μg/mL. In the agar well diffusion assay, S2@ZIF-8 produced an inhibition zone of 12.1 ± 0.35 mm. The antibiofilm activity evaluated at the MIC revealed inhibition rates of 52.6 ± 0.50%**,** 33.5 ± 0.50%, and 29.9 ± 0.17% for S2@ZIF-8, S1@ZIF-8, and pristine ZIF-8, respectively. Furthermore, gene expression analysis demonstrated that S2@ZIF-8 exerted a marked inhibitory effect on *acrB* and *htpX* gene expression, while producing a comparatively moderate suppressive effect on *recA*. **Conclusions:** Overall, S2@ZIF-8 exhibited superior antibacterial and antibiofilm activity among the tested formulations.

## 1. Introduction

Foodborne diseases continue to pose a major global public health challenge, causing an estimated 866 million illnesses and 1.52 million deaths annually while placing a substantial burden on public health systems, food security, and national economies [1]. Among the major bacterial foodborne pathogens, including *Salmonella* spp., *Listeria monocytogenes*, *Campylobacter* spp., and *Escherichia coli*, *E. coli* is recognized as one of the leading bacterial etiological agents responsible for foodborne infections and food contamination worldwide, which poses a persistent threat to public health and the integrity of the food supply chain [2,3]. The rapid emergence and dissemination of antimicrobial-resistant *E. coli* strains have further compromised the effectiveness of conventional antimicrobial therapies, creating significant challenges for food safety management and infection control [4,5]. Moreover, the remarkable capacity of *E. coli* to survive under adverse environmental conditions and establish persistent biofilms on food-contact surfaces significantly enhances its tolerance to disinfectants and antimicrobial agents, thereby facilitating long-term persistence and cross-contamination throughout food-processing environments [6,7]. Collectively, these challenges highlight the urgent need for innovative and effective alternative antimicrobial strategies to mitigate the prevalence of foodborne *E. coli* throughout the food production chain while enhancing food safety and reducing reliance on conventional antimicrobials [8].

However, the successful development and implementation of these alternative antimicrobial strategies require a comprehensive understanding of the adaptive mechanisms that enable foodborne *E. coli* to withstand antimicrobial interventions under the diverse environmental conditions encountered throughout the food production chain. The effectiveness of alternative antimicrobial strategies against foodborne *E. coli* is challenged not only by the bacterium’s ability to survive antimicrobial exposure but also by its highly coordinated stress-response mechanisms, which are activated under diverse environmental and antimicrobial stresses encountered throughout the food production chain, thereby facilitating bacterial adaptation to hostile conditions [9,10]. Among these adaptive mechanisms, bacterial DNA damage response, membrane proteostasis, and multidrug efflux systems constitute key determinants of bacterial survival and stress adaptation [9,11]. In particular, the RecA-mediated SOS response coordinates DNA repair, homologous recombination, and recovery following genotoxic stress, whereas HtpX contributes to membrane protein quality control under stress conditions, and AcrB functions as a major multidrug efflux pump that mediates resistance to a broad spectrum of antimicrobial agents [12,13,14]. Recent evidence has indicated that these systems contribute to antimicrobial resistance, biofilm-associated tolerance, and bacterial persistence by facilitating adaptation to oxidative, environmental, and antibiotic-induced stresses [15,16]. Consequently, antimicrobial strategies capable of disrupting bacterial stress-response pathways, including *recA*-, *htpX*-, and *acrB*-associated mechanisms, may not only enhance antibacterial efficacy but also provide valuable mechanistic insights into bacterial adaptation beyond conventional susceptibility assessments [14].

In addition, natural antimicrobial compounds capable of targeting bacterial survival and biofilm persistence have attracted increasing attention as complementary approaches for controlling foodborne pathogens [16,17]. Silibinin has become a particularly promising antimicrobial candidate due to its broad spectrum of biological activities and favorable safety profile [18]. Silibinin, the major flavonolignan found in *Silybum marianum* seeds, has been shown to have antimicrobial and antibiofilm properties against a variety of bacterial pathogens by preventing bacterial growth, interfering with biofilm formation, and reducing bacterial virulence. When taken as a whole, these biological properties make silibinin a naturally occurring therapeutic compound that may be useful in treating foodborne bacterial infections [19]. Despite these promising antimicrobial properties, the practical application of silibinin remains considerably limited by its extremely poor aqueous solubility, slow dissolution rate, and consequently low bioavailability, all of which substantially compromise its antibacterial efficacy [20].

In order to overcome the physicochemical limitations associated with poorly soluble bioactive compounds, nanotechnology-based drug delivery systems have emerged as practical ways to improve solubility, bioavailability, and antibacterial activity [21]. Among these nanocarriers, zeolitic imidazolate framework-8 (ZIF-8), a zinc-based member of the metal–organic framework (MOF) family, has emerged as a particularly attractive drug delivery platform owing to its high surface area, tunable porosity, high drug-loading capacity, physicochemical stability, and pH-responsive drug release [22,23].

These structural characteristics make ZIF-8 particularly suitable for encapsulation of poorly water-soluble bioactive compounds such as silibinin, to improve drug loading, physicochemical stability and controlled release, which are all crucial for improving antimicrobial performance [24]. In addition to serving as an efficient drug delivery platform, ZIF-8 has been shown to exhibit intrinsic antibacterial activity through multiple mechanisms, including membrane disruption, Zn^2+^ ion release, and reactive oxygen species (ROS)-mediated oxidative stress [25]. Consequently, encapsulating silibinin within a ZIF-8 nanoplatform represents a rational strategy to overcome the physicochemical limitations of the free compound while potentially enhancing its antibacterial and antibiofilm efficacy through the combined advantages of controlled release and the intrinsic antimicrobial properties of ZIF-8 [26].

Silibinin has been incorporated into various nanocarrier systems and ZIF-8 has been extensively investigated as both a drug delivery platform and an antimicrobial nanomaterial. However, the antibacterial efficacy and underlying molecular mechanisms of silibinin-loaded ZIF-8 against foodborne *E. coli* have not been comprehensively investigated [27]. Previous studies have primarily focused on improving the physicochemical properties and antimicrobial performance of silibinin through nanoformulation rather than investigating the bacterial molecular responses induced by this nanoplatforms [28]. In particular, it is still unclear whether silibinin-loaded ZIF-8 can simultaneously inhibit bacterial growth and biofilm formation while modulating the expression of genes associated with stress response, biofilm formation, and antimicrobial resistance, including *recA*, *htpX*, and *acrB*. A thorough understanding of these unresolved mechanisms has become crucial to explain the molecular responses of foodborne *E. coli* against natural compound-loaded nanoplatforms and to facilitate the development of robust, targeted and effective nanotechnology-based antimicrobial strategies for food safety management.

This study, therefore, aimed to investigate the antibacterial, antibiofilm, and molecular effects of silibinin-loaded ZIF-8 (Silibinin@ZIF-8) nanocomposites against foodborne *E. coli*. The antibacterial and antibiofilm activities were evaluated using MIC determination, and crystal violet biofilm assays. Furthermore, the molecular response of *E. coli* to Silibinin@ZIF-8 treatment was investigated by analyzing the expression of selected genes associated with stress response and antimicrobial resistance, such as *recA*, *htpX*, and *acrB*, to provide mechanistic insights into the antibacterial activity of the developed nanocomposite. The findings of this study are expected to provide a scientific foundation for the rational design and development of natural compound-loaded nanoplatforms as effective antimicrobial interventions to control foodborne pathogens, thereby improving food safety and supporting more sustainable food preservation strategies.

## 2. Material and Methods

### 2.1. Synthesis of Silibinin-Loaded ZIF-8 Nanomaterials

ZIF-8 and silibinin-loaded ZIF-8 nanocomposites were synthesized using a one-pot synthesis method according to a previously reported procedure [29]. Briefly, 1.20 g of zinc acetate [Zn(Ac)_2_] was dissolved in 40 mL of deionized (DI) water, while 2.64 g of 2-methylimidazole (2-MIM) was dissolved in 80 mL of methanol. The two solutions were mixed, vortexed, and stirred at room temperature for 4 h. The resulting precipitate was collected by centrifugation at 8000 rpm for 15 min and washed three times with methanol. For the preparation of silibinin-loaded ZIF-8 nanocomposites, the same procedure was followed, except that 10 mg or 20 mg of silibinin was added to the precursor solution prior to mixing, yielding S1@ZIF-8 and S2@ZIF-8, respectively. The silibinin amounts of 10 [30] and 20 mg were selected to provide two distinct initial loading levels while maintaining identical ZIF-8 synthesis conditions, thereby allowing evaluation of the effect of increasing the initial silibinin amount on its incorporation into the ZIF-8-based nanomaterial. These amounts were selected considering comparable initial silibinin quantities previously employed in silibinin-loaded nanocarrier formulations [30,31]. Importantly, the 10 and 20 mg values represent the initial amounts of silibinin introduced during synthesis and not the amounts quantitatively incorporated into the final nanocomposites; the actual incorporation was subsequently evaluated by determination of the encapsulation efficiency. The synthesis procedure is schematically illustrated in Scheme 1.

### 2.2. Characterization

The synthesized pristine ZIF-8, S1@ZIF-8, and S2@ZIF-8 were characterized by Fourier transform infrared spectroscopy (FT-IR; FT/IR-6600, JASCO, Tokyo, Japan) over the spectral range of 400–4000 cm^−1^. The morphology and elemental composition of the samples were investigated using field-emission scanning electron microscopy coupled with energy-dispersive X-ray spectroscopy (FE-SEM/EDX; Quanta 650 FEG, FEI Corporation, Hillsboro, OR, USA). The crystalline structures of the synthesized materials were analyzed by X-ray diffraction (XRD) using a PANalytical EMPYREAN diffractometer (Malvern Panalytical, Almelo, The Netherlands).

#### Determination of Silibinin Loading

The silibinin content of S2@ZIF-8 was determined using UV–Vis spectrophotometry. An appropriate amount of the dried S2@ZIF-8 nanocomposite was treated with 50 µL of 2 M hydrochloric acid to disrupt the ZIF-8 structure and release the incorporated silibinin. The resulting mixture was subsequently diluted to a final volume of 2 mL with ethanol. After complete dissolution, the absorbance of the solution was measured at 288 nm. The amount of silibinin present in S2@ZIF-8 was quantified using a calibration curve prepared with known concentrations of free silibinin under the same analytical conditions. The encapsulation efficiency (EE) of silibinin in the S1@ZIF-8 and S2@ZIF-8 formulations was determined indirectly by measuring the amount of unencapsulated silibinin remaining in the supernatant after centrifugation. Following synthesis, the samples were centrifuged at 8000 rpm for 15 min, and the supernatant was collected. The concentration of free silibinin in the supernatant was quantified using the previously established calibration curve. Encapsulation efficiency was calculated according to the following equation:

$$EE\left(\%\right)=\frac{W_{initial}-W_{free}}{W_{initial}}\times 100$$

where $W_{initial}$ is the initial amount of silibinin added during synthesis and $W_{free}$ is the amount of unencapsulated silibinin detected in the supernatant after centrifugation.

For S1@ZIF-8 and S2@ZIF-8, the initial silibinin amounts were 10 mg and 20 mg, respectively.

### 2.3. Antimicrobial Activity

#### 2.3.1. MIC and MBC Assay

The antibacterial activities of S1@ZIF-8, S2@ZIF-8, and ZIF-8 nanomaterials and free silibinin were determined using the broth microdilution method with resazurin as an indicator [32]. *Escherichia coli* (ATCC 25922), *Escherichia coli* O157:H7 (ATCC 35150), *Salmonella enterica* subsp. *enterica* serovar Typhimurium (ATCC 14028), and *Listeria monocytogenes* (N 7144) were used as the test microorganisms.

Antibacterial activity assays were performed in sterile 96-well microplates. From the stock solutions of S1@ZIF-8, S2@ZIF-8, and ZIF-8, 100 µg/mL was taken and prepared in the respective wells using a two-fold serial dilution method. The bacterial strains were activated by overnight incubation at 37 °C in appropriate culture media. The bacterial suspension was adjusted to the 0.5 McFarland standard (~1–2 × 10^8^ CFU/mL) and subsequently diluted with sterile Mueller–Hinton Broth. Then, 100 µL of the bacterial suspension was added to each corresponding well. The nanomaterials were prepared to obtain final concentrations ranging from 2 to 1024 µg/mL, while the bacterial suspensions were adjusted to achieve a final cell density of approximately 5 × 10^5^ CFU/mL in each well.

Following incubation, 20 µL of sterile freshly prepared resazurin solution was added to each well, and the plates were incubated for an additional 1–3 h. Resazurin turned pink in the presence of metabolically active bacteria, whereas wells in which bacterial growth was inhibited retained the blue color. The minimum inhibitory concentration (MIC) was defined as the lowest sample concentration at which no visible color change occurred (blue wells), indicating the absence of detectable bacterial metabolic activity.

For the determination of the minimum bactericidal concentration (MBC), aliquots were aseptically collected from the wells showing no visible microbial growth in the MIC assay and inoculated onto Mueller–Hinton Agar (MHA) plates. After incubation under appropriate temperature and time conditions, bacterial colony formation was evaluated. The lowest concentration resulting in a 99.9% reduction in viable bacterial count compared with the initial inoculum was defined as the MBC [33].

#### 2.3.2. Agar Well Diffusion Method

The antimicrobial potential of S1@ZIF-8, S2@ZIF-8, and ZIF-8 nanoformulations was investigated in vitro using the agar well diffusion method [34]. In the present study, fresh cultures of the *E. coli* strain exhibiting the lowest MIC value were prepared in sterile 0.85% NaCl solution to obtain homogeneous bacterial suspensions. The cell density was adjusted according to the 0.5 McFarland standard and standardized to approximately 1–2 × 10^8^ CFU/mL. The prepared inocula were spread uniformly onto the surface of Mueller–Hinton Agar (MHA) plates using sterile cotton swabs by the three-direction streaking method. Subsequently, wells with a diameter of 7 mm were punched into the agar, and 100 µL of each sample solution at a concentration of 8 mg/mL was added to the respective wells. For comparison of antimicrobial activity, a Gentamicin disk (Oxoid™ Gentamicin 30 μg; CN) was used as the positive control. The plates were incubated at 37 °C for 18–24 h, after which the inhibition zones were measured in millimeters and evaluated. All experiments were performed in triplicate, and the results were presented as mean values.

### 2.4. Antibiofilm Activity

The antibiofilm effects of S1@ZIF-8, S2@ZIF-8, and ZIF-8 nanoformulations and free silibinin on *E. coli* were evaluated using the crystal violet assay [35]. For biofilm formation, *E. coli* cultures adjusted to the 0.5 McFarland standard and diluted 1:100 in Tryptic Soy Broth (TSB) were distributed into sterile 96-well microplates. The plates were incubated at 37 °C for 24 h to allow bacterial adhesion and the formation of mature biofilms. At the end of the incubation period, the contents of the wells were aspirated to remove planktonic cells, and the wells were gently washed with sterile phosphate-buffered saline (PBS). The established biofilms were then treated with free silibinin, S1@ZIF-8, S2@ZIF-8, and ZIF-8 at concentrations corresponding to 2 × MIC, MIC, and ½ × MIC, followed by a second incubation at 37 °C for 24 h. After treatment, non-adherent bacterial cells were removed, and the remaining biofilms were fixed with methanol to preserve the biofilm matrix. Following fixation, the biofilm layers were stained with 0.5% crystal violet solution. Excess stain was removed, the wells were washed with PBS, and allowed to dry. The crystal violet bound to the adherent biofilm biomass was subsequently dissolved with 95% ethanol, and the absorbance was measured at 570 nm using a microplate reader. All experiments were performed in triplicate, and the percentages of biofilm inhibition were calculated based on the optical density (OD) values.

Antibiofilm rate (%) = (C − S)/C × 100

where C is the control absorbance and S is the sample absorbance.

### 2.5. Gene Expression Analysis 

The expression levels of the *acrB*, *recA*, and *htpX* target genes in *E. coli* treated with S2@ZIF-8 were investigated, using the 16S rRNA gene as the reference gene (Table 1).

Following 24 h treatment of bacterial cultures with the selected compound, total RNA was extracted using TRIzol reagent (Nucleogene Tri-Reagent, NGE-023, İstanbul, Turkey) according to the manufacturer’s instructions. RNA purity was evaluated using a NanoDrop spectrophotometer (DS-11, DeNovix Inc., Wilmington, DE, USA). The RNA concentration was normalized to 200 ng/µL. Subsequently, complementary DNA (cDNA) was synthesized from the isolated RNA samples using the Transcriptor First Strand cDNA Synthesis Kit (Roche, Basel, Switzerland; Cat. No. 04896866001) under the following conditions: 25 °C for 10 min, 55 °C for 30 min, and 85 °C for 5 min. Quantitative real-time PCR was performed using the LightCycler^®^ FastStart DNA Master SYBR Green I kit (Roche, Germany) on a LightCycler^®^ 96 Real-Time PCR System (Roche, Basel, Switzerland). The PCR conditions were as follows: an initial denaturation at 95 °C for 5 min, followed by 40 cycles of 95 °C for 5 s and primer annealing at the optimal temperatures for 30 s. Relative gene expression levels were calculated using the 2^−ΔΔCt^ method [36].

## 3. Results and Discussion

### 3.1. Characterization

#### 3.1.1. FTIR

The FT-IR spectrum of pure silybin exhibited characteristic absorption bands at 3448 cm^−1^, corresponding to phenolic and alcoholic O–H stretching vibrations; 3132–3068 cm^−1^, assigned to aromatic C–H stretching; 2928 cm^−1^, attributed to aliphatic C–H stretching; 1629 cm^−1^, corresponding to conjugated C=O stretching; 1509 and 1466 cm^−1^, assigned to aromatic C=C ring stretching; 1266 cm^−1^, corresponding to phenolic C–O stretching; 1164 and 1081 cm^−1^, attributed to C–O and C–O–C stretching; and 966.1, 822.4, and 734.1 cm^−1^, corresponding to aromatic C–H out-of-plane bending vibrations.

The FT-IR spectrum of ZIF-8 displayed characteristic peaks at 1583 cm^−1^ (C=N stretching), 1436 and 1324 cm^−1^ (imidazolate ring stretching and CH_3_ bending), 1161 and 1010 cm^−1^ (C–N stretching), 843.3, 761.6, and 701.2 cm^−1^ (imidazolate ring bending vibrations), and 420.4 cm^−1^, corresponding to Zn–N stretching.

In the FT-IR spectrum of S1@ZIF-8, characteristic bands were observed at 3135 cm^−1^ (aromatic C–H stretching), 2969 and 2927 cm^−1^ (aliphatic C–H stretching), 1581 cm^−1^ (C=N stretching), 1423 and 1310 cm^−1^ (imidazolate ring stretching and CH_3_ bending), 1146 and 994.8 cm^−1^ (C–N stretching), 822.2, 759.8, and 693.1 cm^−1^ (imidazolate ring bending vibrations), and 420.4 cm^−1^ (Zn–N stretching).

Similarly, the FT-IR spectrum of S2@ZIF-8 exhibited absorption bands at 3135 cm^−1^ (aromatic C–H stretching), 2968 cm^−1^ (aliphatic C–H stretching), 1638 cm^−1^ (C=O stretching), 1590 cm^−1^ (C=N stretching), 1422 and 1310 cm^−1^ (imidazolate ring stretching and CH_3_ bending), 1146 and 994.7 cm^−1^ (C–N stretching), 827.1, 759.7, and 693.1 cm^−1^ (imidazolate ring bending vibrations), and 420.4 cm^−1^ (Zn–N stretching).

Compared with pristine ZIF-8, the C–N stretching band shifted from 1161 cm^−1^ to 1146 cm^−1^ in both S1@ZIF-8 and S2@ZIF-8, indicating that the local chemical environment of the imidazolate linker was altered following silybin incorporation. Furthermore, the characteristic C=O stretching band of silybin, located at 1629 cm^−1^, shifted to 1638 cm^−1^ and became more prominent in S2@ZIF-8, reflecting the higher silybin loading in this formulation. Importantly, the Zn–N stretching vibration remained unchanged at 420.4 cm^−1^ in both silybin-loaded samples, confirming that the crystalline framework of ZIF-8 was preserved after drug loading (Figure 1).

#### 3.1.2. SEM

SEM analysis was performed to investigate the morphology of pristine ZIF-8 and silibinin-loaded ZIF-8 nanoparticles. As shown in Figure 2A, pristine ZIF-8 nanoparticles exhibited a relatively uniform spherical to sub-spherical morphology with smooth surfaces and slight particle agglomeration. The particle size was approximately 100–180 nm. Following silibinin loading (Figure 2B), the overall morphology of the nanoparticles was preserved; however, a remarkable increase in surface roughness was observed. The silibinin-loaded particles displayed a wrinkled and textured outer surface compared with the smooth morphology of pristine ZIF-8. In addition, slight particle enlargement and aggregation were observed after drug loading. These morphological changes suggest the successful incorporation of silibinin into the ZIF-8 framework while maintaining its structural integrity. Similar increases in surface roughness following drug encapsulation have been reported previously for drug-loaded ZIF-8 systems.

#### 3.1.3. EDX

EDX analysis was performed to compare the elemental composition of pristine ZIF-8 and S2@ZIF-8. As shown in Figure 3A, pristine ZIF-8 predominantly contained C, N, O, and Zn, with weight percentages of 46.07%, 9.56%, 1.71%, and 39.69%, respectively. In comparison, S2@ZIF-8 exhibited C, N, O, and Zn contents of 43.78%, 28.32%, 12.59%, and 10.20%, respectively (Figure 3B). Notably, the oxygen content increased markedly from 1.71% in pristine ZIF-8 to 12.59% in S2@ZIF-8. This enrichment in oxygen is consistent with the presence of the oxygen-rich silibinin molecule (C_25_H_22_O_10_) in the resulting material. Concurrent changes in the relative C, N, and Zn contents further indicate modification of the elemental composition following one-pot synthesis in the presence of silibinin. Nevertheless, because EDX provides elemental rather than molecular or structural information, these compositional changes alone cannot unequivocally demonstrate intrapore encapsulation of silibinin. Therefore, the EDX results are considered complementary evidence supporting the formation of the silibinin-containing ZIF-8 material when interpreted together with the other physicochemical characterization and encapsulation-efficiency results.

#### 3.1.4. XRD Analysis

XRD analysis was performed to evaluate the structural characteristics of ZIF-8 following one-pot synthesis in the presence of silibinin. Pristine ZIF-8 exhibited prominent diffraction peaks at 2θ values of 7.28°, 10.34°, 11.04°, 12.67°, 14.65°, 16.41°, 17.98°, and 19.44°, confirming its predominantly crystalline structure. A broad background contribution was also observed in the low-angle region, which may reflect a minor disordered/amorphous fraction or sample-related background scattering. Following silibinin incorporation, S2@ZIF-8 retained several of the major diffraction reflections characteristic of the ZIF-8 framework, with only minor shifts in their 2θ positions. However, changes in relative peak intensities were observed, and the reflection at approximately 2θ ≈ 11° was markedly attenuated and was no longer clearly resolved in the S2@ZIF-8 pattern. This selective attenuation may indicate local structural disorder or changes in crystallinity associated with the incorporation of silibinin during one-pot synthesis. Nevertheless, the persistence of the major characteristic ZIF-8 reflections indicates that the overall crystalline framework was substantially preserved, although some degree of local structural modification occurred upon silibinin incorporation (Figure 4).

#### 3.1.5. Determination of Silibinin Encapsulation Efficiency

The encapsulation efficiencies of S1@ZIF-8 and S2@ZIF-8 were quantitatively determined to evaluate the retention of silibinin within the nanocomposite formulations. The EE values were found to be 77.3 ± 1.2% for S1@ZIF-8 and 80.3 ± 3.1% for S2@ZIF-8, indicating efficient incorporation of silibinin during the one-pot synthesis process. S2@ZIF-8 exhibited a slightly higher mean encapsulation efficiency than S1@ZIF-8. The standard calibration curve for silibinin is provided in the Supplementary Material (Figure S1). Overall, the high EE values obtained for both formulations demonstrate that a substantial proportion of the initially added silibinin was retained in the resulting ZIF-8–based nanocomposites. Although methanol washing was employed to remove non-incorporated silibinin, the presence of a minor fraction of loosely or surface-associated silibinin cannot be completely excluded. Therefore, the calculated EE should be interpreted as an indirect estimate of silibinin association with ZIF-8 rather than definitive evidence of exclusively intrapore encapsulation.

### 3.2. Evaluation of Microbial Assay

#### 3.2.1. Determination of the Antibacterial Activity of S1@ZIF-8, S2@ZIF-8, and ZIF-8 Nanocomposites by MIC and MBC Analyses

The antibacterial activities of S1@ZIF-8, S2@ZIF-8, and ZIF-8 materials against different bacterial pathogens were evaluated by determining their minimum inhibitory concentration (MIC) values using the resazurin-based broth microdilution method (Figure 5). The obtained MIC and minimum bactericidal concentration (MBC) values are presented in Table 2.

Against *E. coli*, the strongest antibacterial activity was observed for the S2@ZIF-8 nanoformulation, with a MIC value of 32 µg/mL. In contrast, the MIC values of both S1@ZIF-8 and ZIF-8 were determined to be 256 µg/mL. For *E. coli* O157:H7, the MIC value of S2@ZIF-8 was 256 µg/mL, whereas the MIC values of S1@ZIF-8 and ZIF-8 were 512 µg/mL.

For the Gram-positive bacterium *L. monocytogenes*, the MIC value was determined to be 512 µg/mL for all three samples, namely S1@ZIF-8, S2@ZIF-8, and ZIF-8. Similarly, in experiments performed against *S. typhimurium*, all nanoformulations exhibited MIC values of 512 µg/mL. In contrast, the MIC value of free silibinin was determined to be 256 µg/mL for *E. coli* and 512 µg/mL for *E. coli* O157:H7, *L. monocytogenes*, and *S. typhimurium*. Furthermore, the MBC values obtained for the tested bacterial strains ranged from 64 to >1024 µg/mL. These findings indicate that the nanoformulations exhibited concentration-dependent bactericidal activity against the tested bacterial pathogens.

Foodborne diseases remain a major global public health and economic concern. *Salmonella enterica* serovar Typhimurium, pathogenic *E. coli* (e.g., O157:H7), and *L. monocytogenes* are among the most persistent and life-threatening pathogens in the food industry [37,38,39]. Therefore, the development of novel antimicrobial approaches capable of preventing surface-mediated cross-contamination is essential for improving food safety. Nguyen et al. synthesized three different nanoformulations, namely OEO-Ag-ZIF-8-HA, OEO-Fe-ZIF-8-HA, and OEO-HA, by incorporating oregano essential oil (OEO), hyaluronic acid (HA), and metal-doped (Ag and Fe) ZIF-8. In their study, the MIC value against *L. monocytogenes* was determined to be 875 µg/mL for OEO-Fe-ZIF-8-HA, whereas MIC values of 1250 µg/mL were reported for both OEO-Ag-ZIF-8-HA and OEO-HA [40]. In another study, copper-doped ZIF-8 nanoparticles coated with hyaluronic acid were synthesized. Against *S. typhimurium*, the MIC values were reported as 1500 µg/mL for ZIF-8, 750 µg/mL for Cu-ZIF-8, 1250 µg/mL for ZIF-8@HA, and 500 µg/mL for Cu-ZIF-8@HA. Against *E. coli* O157:H7, the MIC values were reported as 2000 µg/mL for ZIF-8, 1500 µg/mL for Cu-ZIF-8, 1250 µg/mL for ZIF-8@HA, and 1000 µg/mL for Cu-ZIF-8@HA [41]. Abdelkhalek et al. reported that phosphomolybdic acid-loaded ZIF-8 (PMA@ZIF-8) exhibited a MIC value of 375 µg/mL against *E. coli*, whereas pristine ZIF-8 showed a MIC value of 750 µg/mL [42].

The bactericidal activity of ZIF-8 has been attributed to the synergistic effects of the controlled release of Zn^2+^ ions and oxidative stress induced by ROS. Released Zn^2+^ ions inhibit bacterial enzyme activities and increase cell membrane permeability, whereas ROS generated by ZIF-8 induce oxidative damage to proteins, lipids, and nucleic acids, thereby accelerating bacterial cell death [43]. The antimicrobial activity of silibinin has been associated with multiple mechanisms, including inhibition of DNA replication, regulation of gene expression, disruption of bacterial cell membrane integrity, and reduction of bacterial motility. In addition, silibinin has been reported to enhance ROS production and suppress quorum sensing systems, thereby reducing biofilm formation [44].

Overall, the S2@ZIF-8 nanoformulation exhibited markedly higher antibacterial activity, particularly against *E. coli*. The low MIC value of S2@ZIF-8 suggests that the active component incorporated into the ZIF-8 structure exerts a synergistic effect, thereby enhancing bacterial growth inhibition. In contrast, the higher MIC values observed against *E. coli* O157:H7, *L. monocytogenes*, and *S. typhimurium* may be attributed to differences in bacterial cell wall characteristics, metabolic properties, and nanoformulation–bacteria interactions among these microorganisms.

#### 3.2.2. Evaluation of Agar Well Diffusion Method

According to the MIC results, *Escherichia coli*, identified as the most susceptible bacterial strain, was selected to evaluate the antimicrobial activities of S2@ZIF-8, S1@ZIF-8, and ZIF-8 using the agar well diffusion method. Based on the obtained results, the S2@ZIF-8-treated group produced an inhibition zone with a diameter of 12.1 ± 0.35 mm, whereas S1@ZIF-8 exhibited an inhibition zone of 11.0 ± 0.28 mm. In contrast, no inhibition zone was observed for the pristine ZIF-8 sample on the agar medium. The gentamicin disk used as the positive control produced an inhibition zone of 22.0 ± 0.50 mm (Figure 6).

The larger inhibition zone produced by the S2@ZIF-8 nanoformulation compared with S1@ZIF-8 suggests that differences in the amount of the incorporated active component and/or its release behavior resulted in more effective suppression of bacterial growth. Consistent with its low MIC value (32 µg/mL), the more pronounced diffusion effect exhibited by S2@ZIF-8 indicates that the nanoformulation may interact more efficiently with *E. coli* cells. Overall, these findings demonstrate that both S2@ZIF-8 and S1@ZIF-8 nanoformulations exhibited enhanced antibacterial performance compared with pristine ZIF-8, suggesting that the nanoformulation strategy can improve the biological activity of the incorporated active component.

#### 3.2.3. Evaluation of Antibiofilm Activity

The antibiofllm effects of S1@ZIF-8, S2@ZIF-8, and ZIF-8 formulations and free silibinin on pre-formed biofilm by *E. coli* were investigated using the crystal violet assay at three different concentrations: 2 × MIC, 1 × MIC, and ½ × MIC. The results demonstrated that all three formulations inhibited biofilm formation in a concentration-dependent manner (Figure 7). The strongest antibiofilm activity was observed at the 2 × MIC, where S2@ZIF-8, S1@ZIF-8, free silibinin and ZIF-8 exhibited biofilm inhibition rates of 67.2 ± 0.40%, 46.1 ± 0.35%, 44.7 ± 0.62% and 36.1 ± 0.32%, respectively. Although the inhibition rates decreased when the concentration was reduced to the MIC level, biofilm formation remained significantly suppressed. At the MIC, the inhibition rates were 52.6 ± 0.50%, 33.5 ± 0.50%, 31.4 ± 0.95% and 29.9 ± 0.17% for S2@ZIF-8, S1@ZIF-8, and ZIF-8, respectively. At the lowest concentration (1/2 × MIC), antibiofilm inhibition rate was further reduced for all formulations.

Extracellular polymeric substances (EPS) are the major structural components of biofilms and play a critical role in bacterial adhesion to surfaces, maintenance of biofilm integrity, and protection against adverse environmental conditions. The protective EPS matrix limits the penetration of antimicrobial agents into the biofilm, making the eradication of mature biofilms considerably more difficult than the inhibition of planktonic bacteria. Consequently, biofilm-associated infections remain a major challenge for effective prevention and treatment [45].

Previous studies have demonstrated that ZIF-8-based nanocomposites effectively inhibit biofilm formation by various bacterial strains [46,47]. In the present study, silibinin-loaded ZIF-8 inhibited *Escherichia coli* biofilm formation in a concentration-dependent manner. Among the tested formulations, S2@ZIF-8 exhibited the highest antibiofilm activity, indicating that incorporation of silibinin into the ZIF-8 framework enhanced its ability to suppress biofilm development. Overall, these findings suggest that S2@ZIF-8 is a promising candidate for preventing microbial contamination on food-contact surfaces and controlling biofilm formation.

### 3.3. Evaluation of Gene Expression Analysis

According to the log_2_ fold change analysis normalized to the untreated control group, treatment with S2@ZIF-8 resulted in the downregulation of all investigated target genes in *E. coli* (Figure 8). The log_2_ fold change analysis normalized to the untreated control group demonstrated a downregulation of all target genes in *E. coli* following treatment with S2@ZIF-8. The most pronounced reduction was observed for the *acrB* gene, with a log_2_ fold change of −5.234, corresponding to a decrease in expression to approximately 2.66% of the untreated control level. The *htpX* gene also exhibited substantial downregulation, with a log_2_ fold change of −2.158, representing an approximately 4.46-fold reduction in expression. In contrast, the recA gene showed the least pronounced response, with a log_2_ fold change of −0.558, corresponding to an approximate 32% decrease in expression relative to the control. Collectively, these findings indicate that S2@ZIF-8 exerts a marked inhibitory effect on *acrB* and *htpX* gene expression, while producing a comparatively moderate suppressive effect on recA.

In the present study, three target genes were investigated in *E. coli* following treatment with S2@ZIF-8. The *acrB* gene encodes the energy-dependent transporter component of the AcrAB–TolC efflux pump in *E. coli*. This system is responsible for the extrusion of toxic compounds from bacterial cells and plays a crucial role in enhancing bacterial tolerance to antimicrobial agents. Swick et al. evaluated the expression of the *acrA* and *acrB* genes in clinical *E. coli* isolates and demonstrated that their overexpression was associated with fluoroquinolone resistance and multidrug resistance [48]. In relation to our findings, Wang et al. investigated a Ag nanoparticle-containing ZIF-8-NH_2_ system against *E. coli*. RNA-seq analysis revealed the downregulation of genes associated with DNA repair, hydrogen peroxide response, and ROS response, together with disruption of membrane transport systems and a reduction in bacterial DNA content [49]. These findings are consistent with the marked downregulation of *acrB* observed in the present study (−5.234 log_2_ fold change, corresponding to an approximately 37.6-fold reduction), suggesting suppression of bacterial membrane transport and defense systems. Furthermore, the simultaneous downregulation of *acrB* and *htpX* may indicate impairment of bacterial membrane-associated protective mechanisms.

The *recA* gene plays a central role in maintaining genomic integrity in bacteria. The RecA protein is essential for homologous recombination and DNA repair and is known to initiate the SOS response by binding to single-stranded DNA following DNA damage. Therefore, the reduced expression of *recA* observed in the present study suggests suppression of the bacterial SOS response and DNA repair capacity [50]. In a recent study, Lin et al. demonstrated that treatment of *E. coli* O157:H7 with K_6_[P_2_Mo_18_O_62_] increased both recA mRNA and RecA protein levels, which was associated with DNA fragmentation [51]. In contrast, treatment with silibinin-loaded ZIF-8 in the present study resulted in downregulation of recA expression by −0.558 log_2_ fold change, corresponding to an approximately 1.47-fold reduction. This relatively moderate decrease may be associated with suppression of the SOS response, reduced overall transcriptional activity, or alterations in the cellular response depending on the treatment duration, suggesting inhibition of recA transcription. Similarly, Şener et al. reported that olivetol, a natural phenolic compound derived from lichens, simultaneously suppressed the expression of *recA*, *sulA*, and *lexA* in *E. coli* O157:H7, with 800 μM olivetol reducing recA expression by approximately 83% [52].

Sakoh et al. reported that the htpX gene participates in membrane protein quality control by removing damaged membrane proteins in the cytoplasmic membrane of *E. coli* [12]. In the present study, htpX expression decreased by −2.158 log_2_ fold change, corresponding to an approximately 4.46-fold reduction. In contrast, Kirchner et al. reported that exposure of *E. coli* O157:H7 to a quaternary ammonium compound-based disinfectant resulted in a 1.59 log_2_ fold increase in *htpX* expression, which was interpreted as a bacterial cell envelope and membrane stress response [53]. The observed −2.158 log_2_ fold change in *htpX* expression in the present study suggests that S2@ZIF-8 may not only induce membrane stress but also impair the bacterial capacity for membrane protein quality control and the removal of damaged membrane proteins. Furthermore, Wang et al. reported that, in addition to htpX, several protein quality control and heat-shock-related genes, including *dnaK*, *dnaJ*, *htpG*, *hslU*, and *hslV*, were also significantly downregulated following treatment with the Ag nanoparticle-containing ZIF-8-NH_2_ system. The authors suggested that this response was associated with impaired repair or removal of misfolded proteins and a broader suppression of the bacterial stress response [49].

## 4. Conclusions

In this study, ZIF-8 and silibinin-loaded ZIF-8 nanocomposites were successfully synthesized and characterized using FT-IR, SEM, EDX, and XRD analyses. The physicochemical characterization supported the association of silibinin with the ZIF-8-based material, while SEM observations revealed morphological changes following synthesis in the presence of silibinin. XRD analysis further indicated substantial preservation of the principal crystalline ZIF-8 framework, although changes in relative peak intensities suggested some degree of local structural modification.

The synthesized nanocomposites exhibited antibacterial activity against *E. coli*, *E. coli* O157:H7, *S. Typhimurium*, and *L. monocytogenes*. Among the tested formulations, S2@ZIF-8 demonstrated the strongest antibacterial activity against *E. coli*, with an MIC value of 32 μg/mL, compared with 256 μg/mL for pristine ZIF-8. S2@ZIF-8 also exhibited pronounced antibiofilm activity against *E. coli*. Furthermore, gene expression analysis showed downregulation of *recA*, *acrB*, and *htpX*, suggesting that the antibacterial effects of S2@ZIF-8 may involve modulation of bacterial responses associated with DNA repair, multidrug resistance, and cellular stress.

Overall, S2@ZIF-8 exhibited superior antibacterial and antibiofilm activity among the tested formulations. Although the higher initial amount of silibinin used for S2@ZIF-8 may have contributed to its enhanced activity, the present data do not establish a direct quantitative relationship between silibinin loading and antibacterial efficacy. The observed effects are therefore more appropriately attributed to the combined influence of silibinin association with ZIF-8 and the resulting physicochemical characteristics of the nanocomposite. These findings highlight S2@ZIF-8 as a promising nanoplatform for further investigation as an alternative antimicrobial system against pathogenic *E. coli*.

## Figures and Tables

**Scheme 1:**
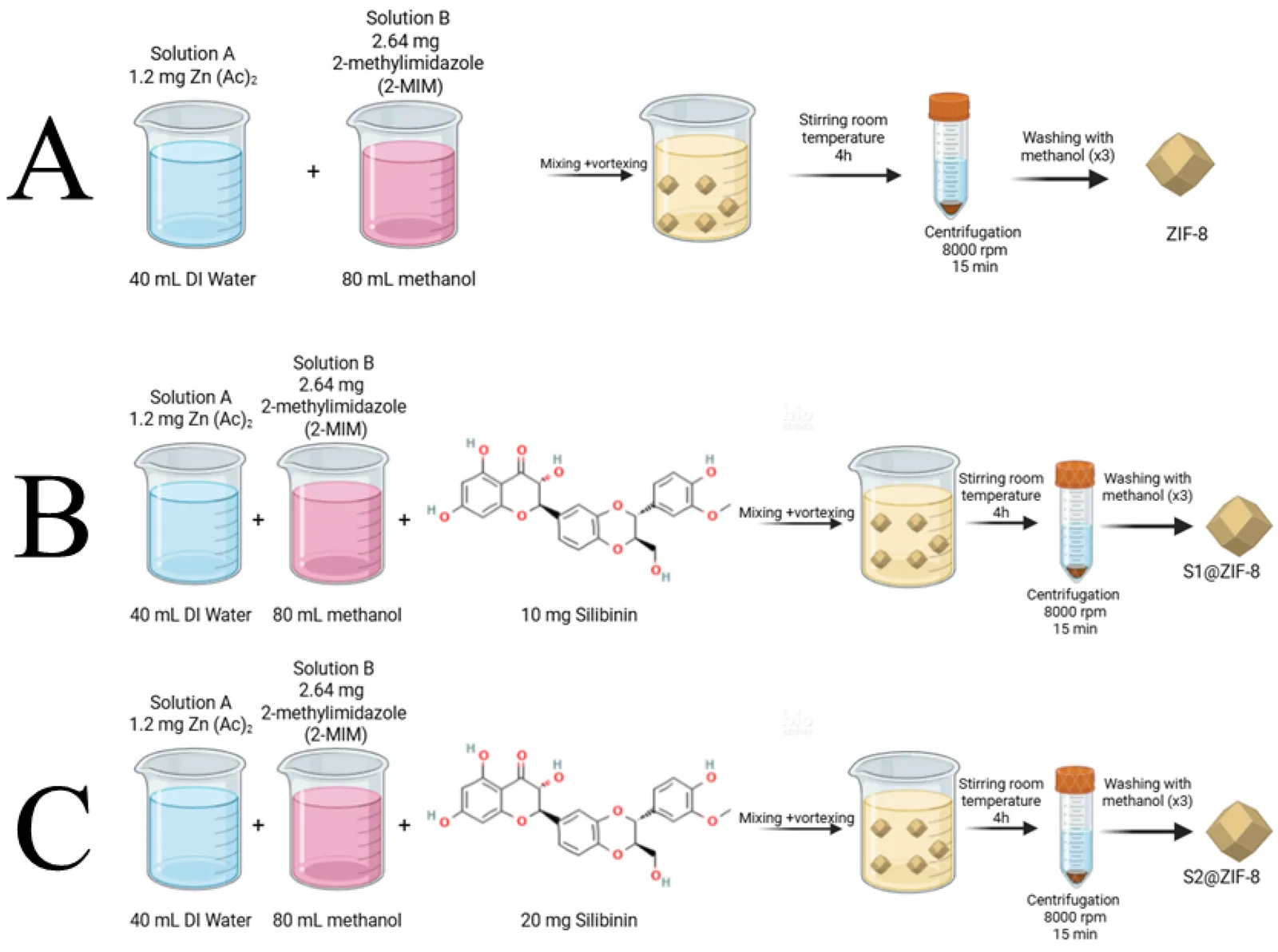
Schematic illustration of the synthesis of (**A**) pristine ZIF-8; (**B**) S1@ZIF-8 using 10 mg of silibinin; and (**C**) S2@ZIF-8 using 20 mg of silibinin.

**Figure 1:**
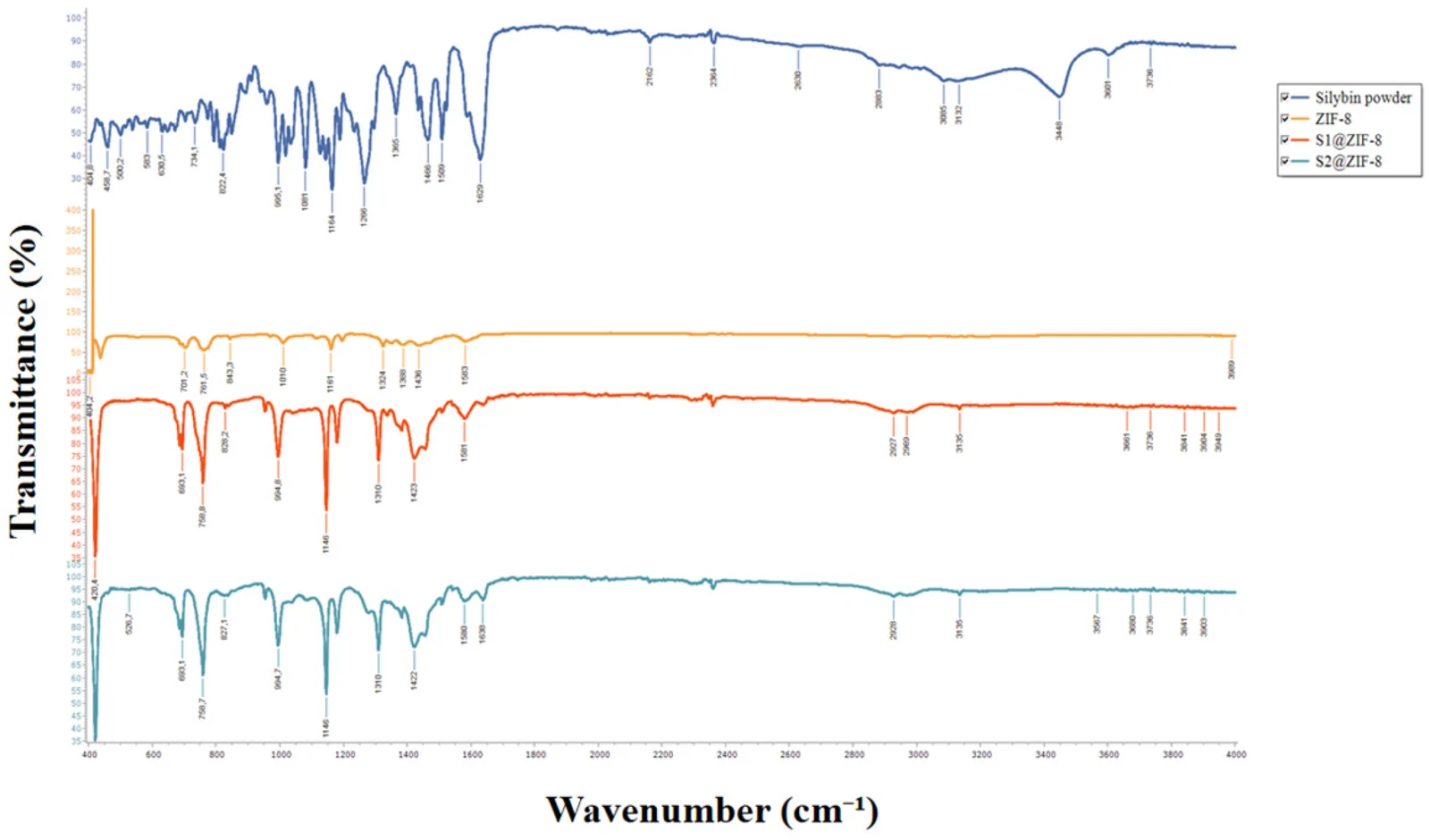
FTIR spectra of Silibinin powder, ZIF-8, S1@ZIF-8 and, S2@ZIF-8.

**Figure 2:**
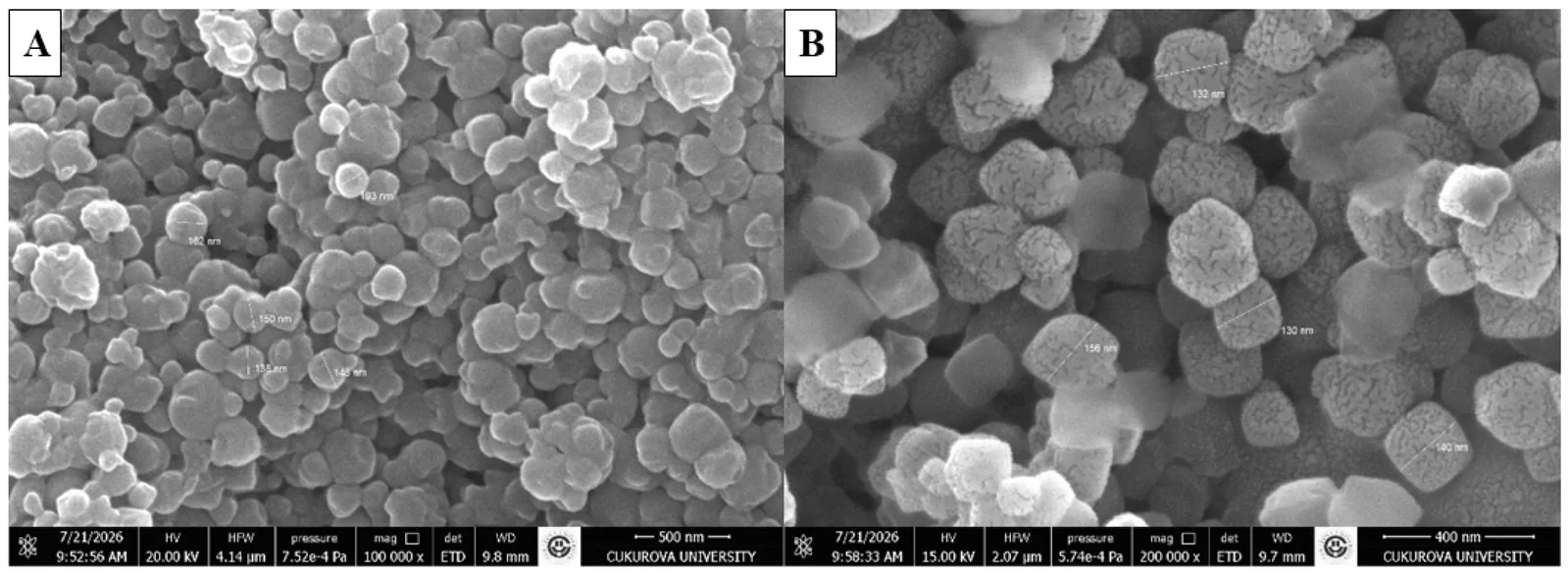
Representative SEM micrographs of (**A**) pristine ZIF-8 nanoparticles showing smooth and uniform spherical morphology and (**B**) silibinin-loaded ZIF-8 nanoparticles exhibiting increased surface roughness and a wrinkled texture after drug loading while preserving the overall particle morphology.

**Figure 3:**
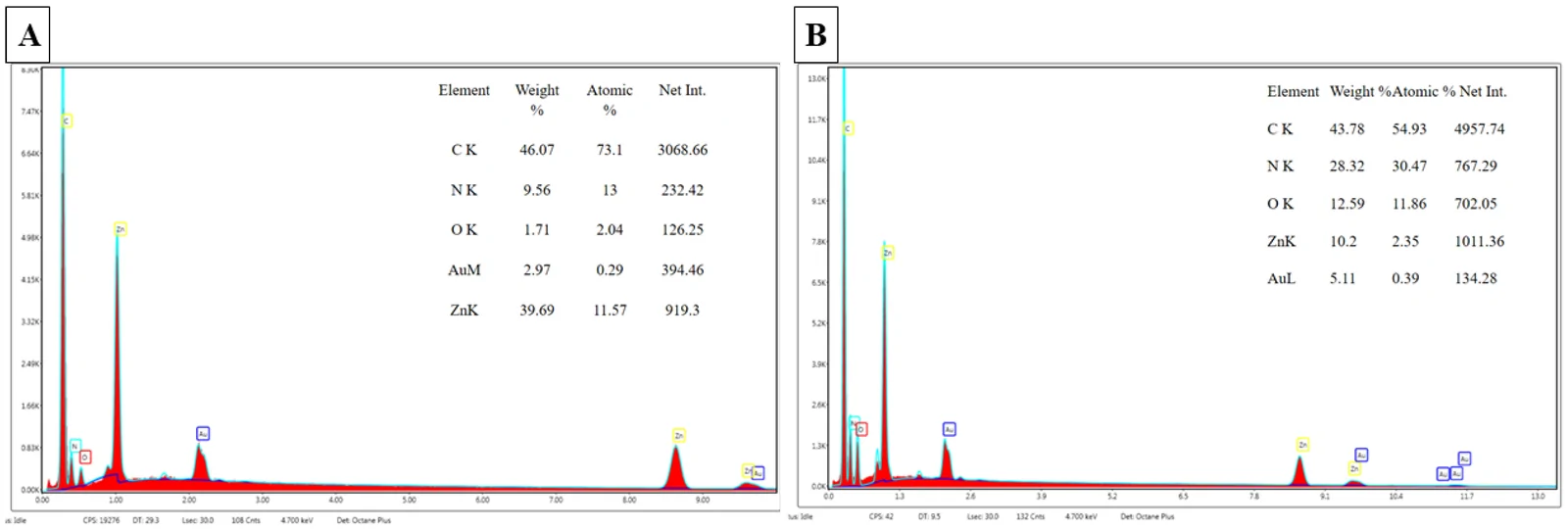
EDX analysis of ZIF-8 (**A**) and S2@ZIF-8 (**B**). The X-axis represents energy levels in keV, while the Y-axis shows the intensity in counts.

**Figure 4:**
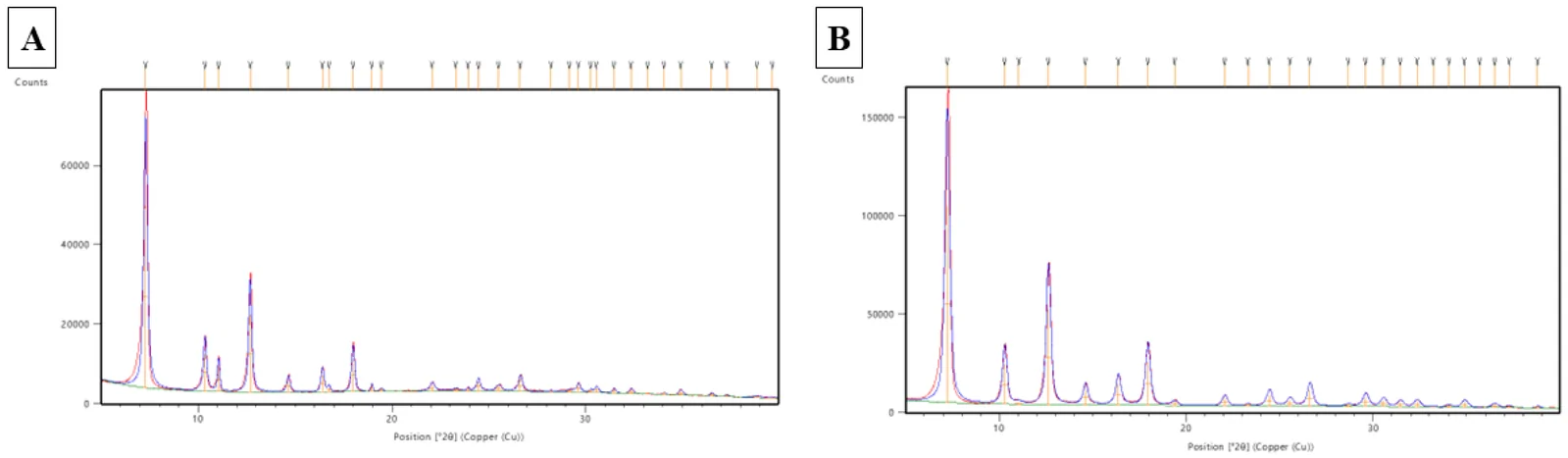
X-ray diffraction (XRD) patterns of (**A**) pristine ZIF-8 and (**B**) S2@ZIF-8. The retention of the characteristic diffraction peaks of ZIF-8 in S2@ZIF-8 indicates preservation of the principal crystalline ZIF-8 structure following one-pot synthesis in the presence of silibinin.

**Figure 5:**
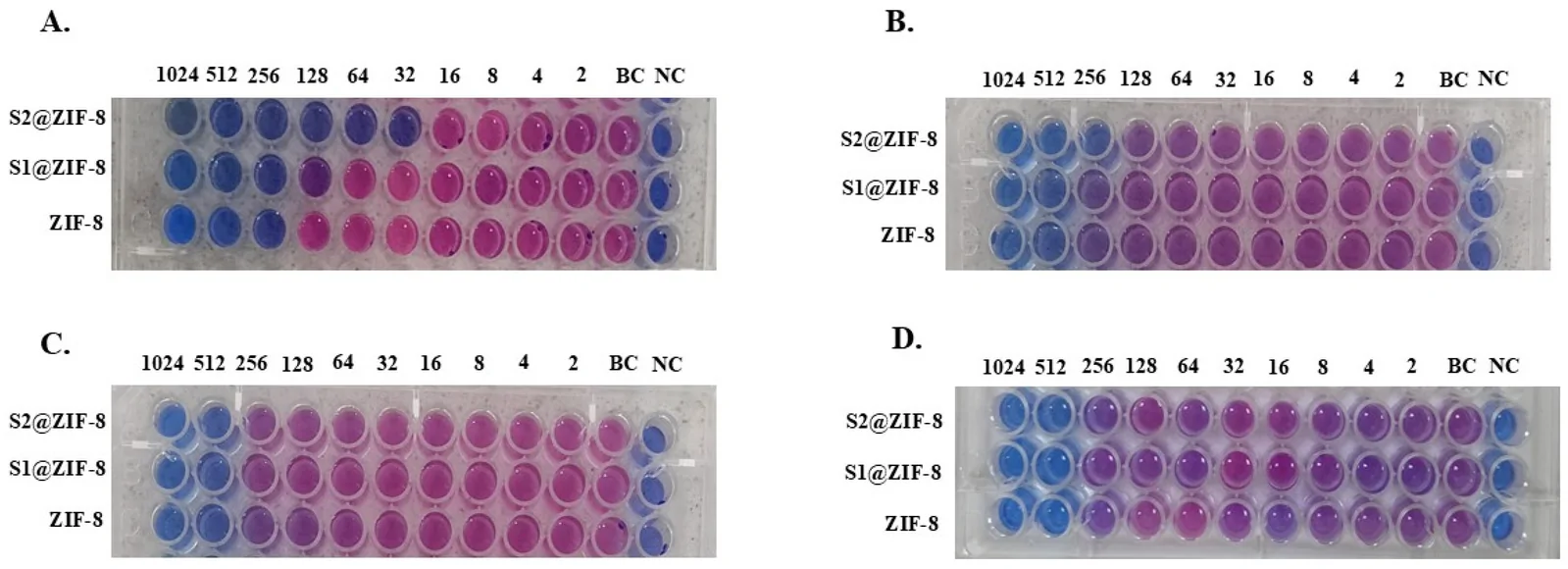
Resazurin MIC assay for (**A**). *E. coli*, (**B**). *E. coli* 0157:H7, (**C**) *S. typhimurium*, and (**D**). *L. monocytogenes*.

**Figure 6:**
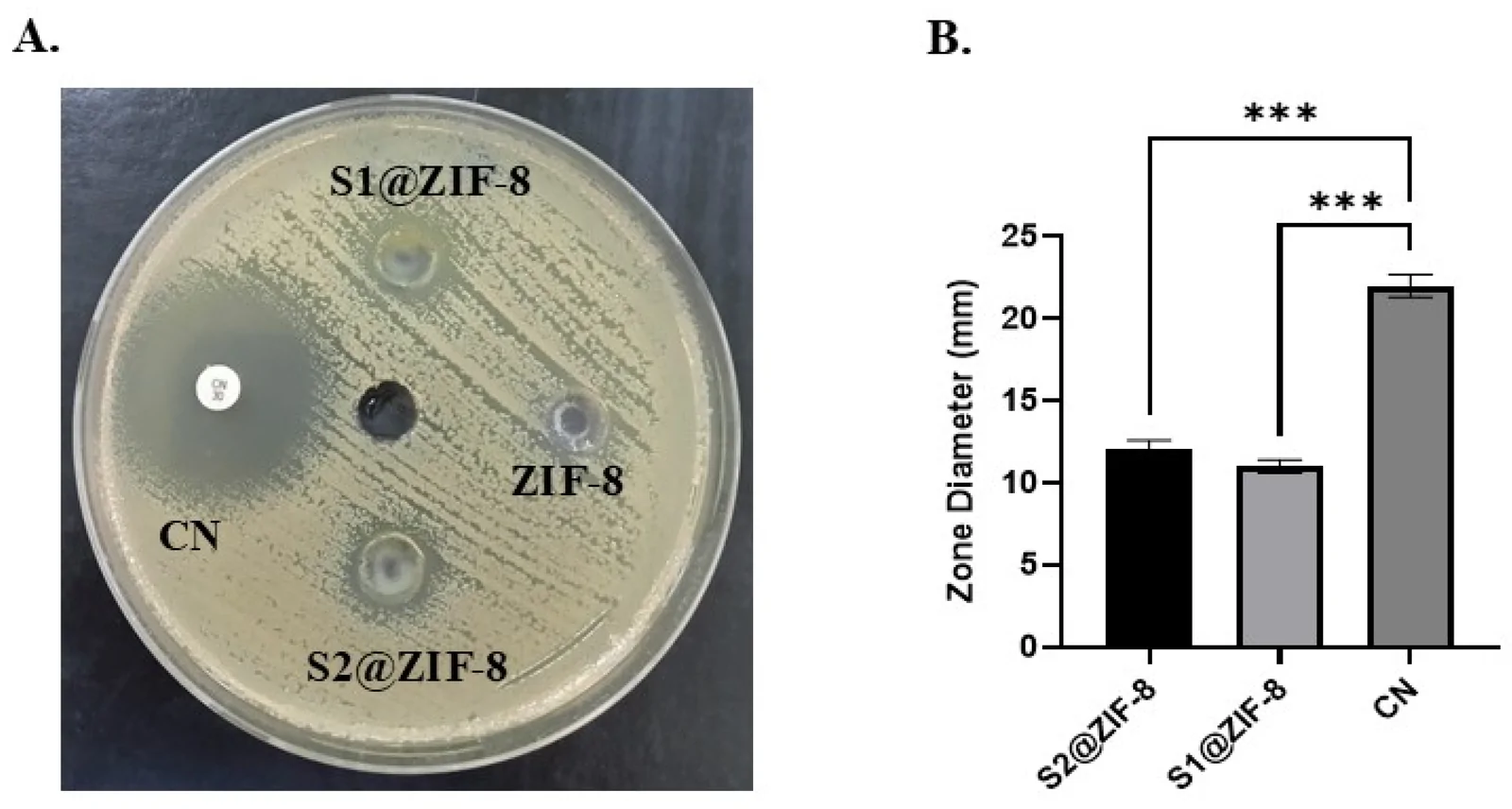
Inhibition zone diameters (mm) of compounds S2@ZIF-8, S1@ZIF-8, and ZIF-8 determined by the agar well diffusion method: (**A**) overall inhibition zones, (**B**) antibacterial activity against *E. coli*. Data are presented as mean ± SD (*n* = 3). *** *p* < 0.001 were considered statistically significant.

**Figure 7:**
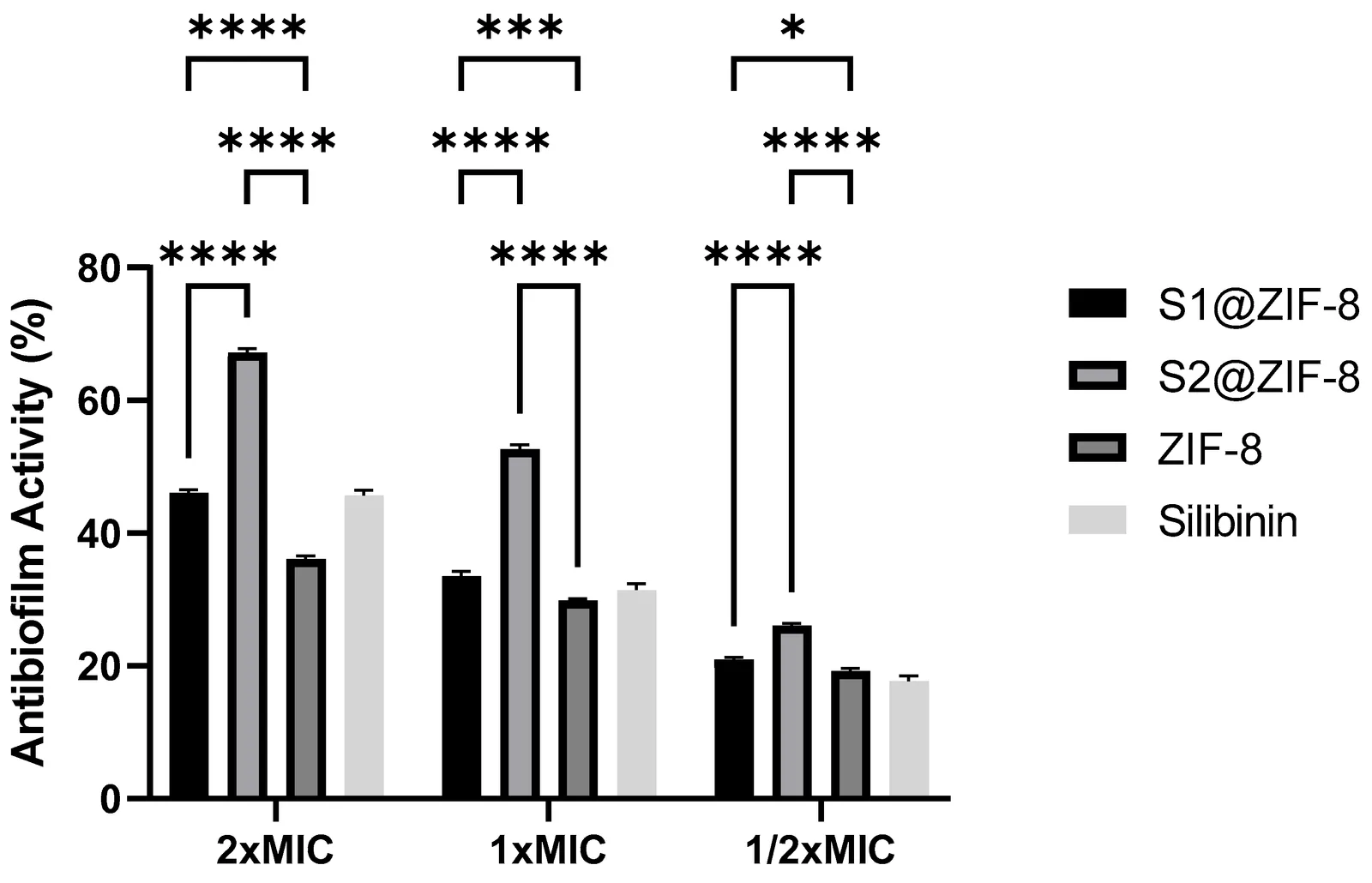
Antibiofilm activity S1@ZIF-8, S2@ZIF-8, ZIF-8, and free silibinin. Data are presented as mean ± SD (*n* = 3). * *p* < 0.05, *** *p* < 0.001, and **** *p* < 0.0001 were considered statistically significant.

**Figure 8:**
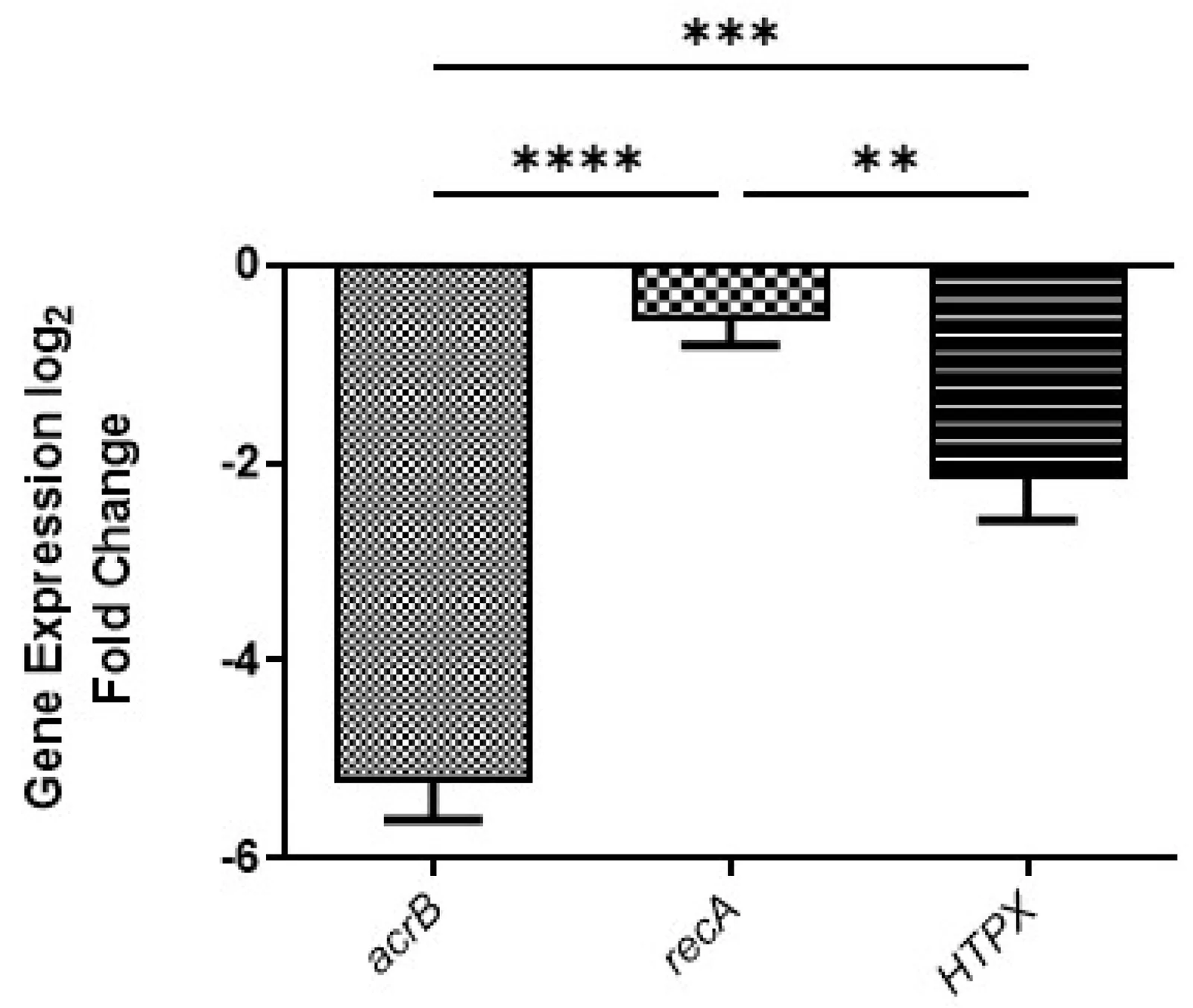
qRT-PCR analysis of gene expression after treatment with S2@ZIF-8. Values represent mean ± SD (*n* = 3). ** *p* < 0.01, *** *p* < 0.001, and **** *p* < 0.0001 were considered statistically significant.

**Table 1:** Gene-specific primer sequences used for qPCR analysis.

Genes	Primers
*acrB*	F GGTCGATTCCGTTCTCCGTTA
	R CTACCTGGAAGTAAACGTCATTGGT
*recA*	F: CAGGCAGTTGCATTCGCTTT
	R: TCTACGGCGAACTGGTTGAC
*HtpX*	F: GGCAACCCGCTGATCTACTT
	R: AGCGCGGCAATCATTTTCTC
16sRNA	F: CGTTACCCGCAGAAGAAGC
	R: GTGGACTACCAGGGTATCTAATCC

**Table 2:** The MIC and MBC values (µg/mL) of the nanoformulations against pathogenic bacteria.

Bacterial Strain	S1@ZIF-8		S2@ZIF-8		ZIF-8		Silibinin
	MIC	MBC	MIC	MBC	MIC	MBC	MIC MBC
*E. coli*	256	512	32	64	256	512	256 1024
*E. coli* O157:H7	512	1024	256	512	512	1024	512 1024
*S. typhimurium*	512	>1024	512	1024	512	>1024	512 1024
*L. monocytogenes*	512	>1024	512	1024	512	>1024	512 >1024

## Data Availability

The datasets used and/or analyzed during the current study are available from the corresponding author upon reasonable request.

## Supplementary Materials

The following supporting information can be downloaded at https://www.mdpi.com/article/10.3390/antibiotics15100951/s1, Figure S1: Standard calibration curve of silibinin over the concentration range of 0–25 mg/mL.
