# Supplementary material for: Synthesis and Characterization of Silibinin-Loaded ZIF-8 Nanocomposites and Their Antibacterial Activity Against Escherichia coli: Insights into acrB, recA, and htpX Gene Expression
Source: Antibiotics (Basel). 2026 Sep 24;15(10):951. doi: 10.3390/antibiotics15100951 (PMC13700648; doi:10.3390/antibiotics15100951)
Supplement: Supplementary file 1 [file antibiotics-15-00951-s001.zip › antibiotics-4507255-supplementary.pdf]

Supplementary Material

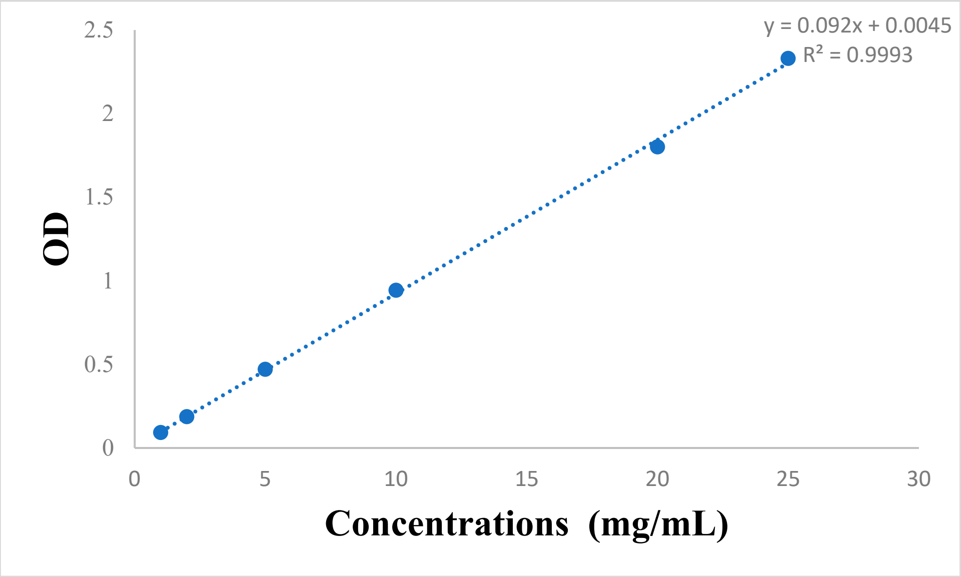

Figure S1. Standard calibration curve of silibinin over the concentration range of 0–25 mg/mL.
